# Populations and Host/Non-Host Plants of Spittlebugs Nymphs in Olive Orchards from Northeastern Portugal

**DOI:** 10.3390/insects11100720

**Published:** 2020-10-21

**Authors:** María Villa, Isabel Rodrigues, Paula Baptista, Alberto Fereres, José Alberto Pereira

**Affiliations:** 1 Centro de Investigação de Montanha (CIMO), ESA, Instituto Politécnico de Bragança, Campus de Santa Apolónia, 5300-253 Bragança, Portugal; mariavillaserrano@gmail.com (M.V.); irodrigues@ipb.pt (I.R.); pbaptista@ipb.pt (P.B.); 2 Instituto de Ciencias Agrarias, Consejo Superior de Investigaciones Científicas, ICA-CSICdpdo, 28006 Madrid, Spain; a.fereres@csic.es

**Keywords:** *Xylella fastidiosa*, Aphrophoridae, *Philaenus spumarius*, *Neophilaenus* sp., development, ground-cover

## Abstract

**Simple Summary:**

*Xylella fastidiosa* is a serious fitopathogenic bacteria which causes severe problems in different crops and ornamental plants. This plant disease is transmitted by insect vectors being spittlebugs the most important in Europe. They are polyphagous and during their young stages feed on herbs, therefore usual recommendations for the reduction of spittlebug populations in perennial crops include the herbaceous ground cover removal. Nevertheless, this practice is undesirable in sustainable agriculture. Thus, in this work the goal was to identify vector species and their young stages preferred/not preferred plants in natural ground covers from olive groves. The study area was located in the northeast of Portugal, a region at risk of infection with *X. fastidiosa*. Several plants were identified as food resources for spittlebugs while some abundant plants presented low numbers of spittlebugs, providing with a new insight about potential plants for integrating ground covers without favoring the disease.

**Abstract:**

The Aphrophoridae family contains important vectors of *Xylella fastidiosa*, a serious bacterial plant disease. In olive orchards, nymphs usually feed on the ground-cover vegetation. However, detailed information about their populations and host/non-host plants in some regions threatened by *Xylella*, such as the northeast of Portugal, is very limited. The goal of our work was to identify the vector species, nymphal development period, and their host and non-host herbaceous plants in olive orchards from northeastern Portugal. Ground-cover plant species hosting or not hosting nymphs were identified during the spring of 2017 to 2019 in olive orchards. Nymphal development period, nymph aggregation, and nymph’s preferred feeding height of the ground-cover plants were recorded. The most abundant Aphrophoridae species was *Philaenus spumarius* followed by *Neophilaenus* sp. Nymphs developed from April to early May and showed a low number of individuals per foam (generally between one and three). They preferred the middle part of the plants. *Philaenus spumarius* feeds preferentially on Asteraceae and Fabaceae, and *Neophilaenus* sp. on Poaceae. Some abundant plants, such as *Bromus diandrus*, *Astragalus pelecinus*, *Chrysanthemum segetum*, *Trifolium* spp., Caryophyllaceae, and Brassicaceae, were barely colonized by Aphrophoridae nymphs. This knowledge is essential for the selection of the species composition of ground-cover vegetation to minimize the presence of vectors of *X. fastidiosa* in olive groves.

## 1. Introduction

*Xylella fastidiosa* Wells is a bacterium that causes severe diseases in many plant species. It affects several important crops (such as olives, grapes, almonds, and citrus), and ornamental and wild plants, threatening economically important plants and landscapes [1]. In 2013, thousands of hectares of olive orchards were devastated and *X. fastidiosa* was identified [2]. Subsequently, it has been detected in several plants and different European countries, such as France, Spain, and Portugal [3].

Xylem-sap feeding insects are the means of *X. fastidiosa* transmission. These insects belong to the suborder Auchenorrhyncha. The family Cicadellidae—leafhoppers (subfamily Cicadellinae—sharpshooters) and the superfamily Cercopoidea (families Aphrophoridae—spittlebugs, Cercopidae—froghoppers, and Clastropteridae) are the main groups of vectors [4]. In Europe, only three Aphrophoridae species have been identified as vectors, *Philaenus spumarius* (L., 1758), *P. italosignus* Drosopoulos & Remane (2000) and *Neophilaenus camprestris* (Fallén, 1805) [5,6]. *Philaenus spumarius* was the first vector identified in Europe [5,7] and is still by far the most abundant [8,9].

Knowledge about the diversity, biology, and population dynamics of potential vectors in regions at risk of infection is paramount to establish strategies to control and/or prevent the spread of this bacterium. In general, the available information for *P. spumarius* is more complete than for the other vectors [10]. *Philaenus spumarius* and *N. campestris* are univoltine and polyphagous species. The first feeds mostly on dicotyledonous and *N. camprestris* on monocotyledoneus [9,11,12,13]. Nymphs of both species hatch in the early spring, crawl to a succulent part of the plant and feed on the sap, developing five nymphal stages during 4 to 8 weeks until molting into adults [13,14]. The period for nymph development varies according to environmental conditions of locations, such as altitude or temperature [3,9,12,13,14,15]. Generally, Aphrophoridae nymphs feed on herbaceous plants occurring within cultivated crops and non-crop habitats, particularly in meadows [16]. Aphrophoridae nymphs excrete spittle bubbly masses or foam through the anus, which surrounds them and produces a constant moist environment [14,17]. Several potential functions, such as protection from dehydration, natural enemies, damaging radiation [17,18] or many pesticides [19], are attributed to this foam. Adults jump and fly, and feed on many plant species but do not produce foam [14,20]. They usually remain in the field until the foliage is removed or dried out during the summer, and then migrate to alternate woody hosts [12,13,14,15,21]. Egg maturation of *P. spumarius* occurs during the first half of August (in colder regions from central-northern Europe) or mid-September and beginning of October (in more southern regions, November in Spain) [3,22,23]. Then, they start the oviposition on herbaceous plants (generally Poaceae) [14]. Eggs of both species overwinter until the following spring [16]. Most adults die during winter.

Although several studies addressed Aphrophoridae diversity and biological aspects in Europe [16,23,24,25,26,27], this knowledge has been insufficient to contain the *X. fastidiosa* outbreak on the continent. Subsequently, a relevant effort has been made to satisfy the knowledge needs and currently new information is available on host plant preference for nymphs and adults, the life cycle, and population dynamics [9,13,15,28]. However, due to the wide geographical rage (from Northern to Southern Europe and North America), the knowledge on the ecology and phenology of the species in the different areas is limited [3]. Additionally, most detailed information about host plants of nymphs and adults of *P. spumarius* comes from the USA [3] and Italy [9,13]. Recent works addressed the effect of several factors driving its spatial distribution in Europe. For example, in Italy, Santoiemma et al. [29] found that *P. spumarius* abundance and presence was positively influenced by olive groves in the landscape and negatively by vineyards at a small spatial scale (125–250 m), and that landscapes at higher elevations dominated by olive orchards are more likely to attract *P. spumarius*. In olive orchards, it has been suggested that *P. spumarius* nymphs develop in the ground-cover vegetation in spring, during summer they move from the drying vegetation to the olive canopy and woody alternative hosts (mainly *Quercus* spp. in some regions of Italy), and in early autumn adults return to the regrowth ground vegetation for oviposition [3,12,13,15]. *Neophilaenus campestris* disperse in the summer looking for trees and shrubs that offer food and shelter until the time of oviposition in the fall [12].

In Italy, in orchards infected with *X. fastidiosa*, *P. spumarius* adults were observed on olive trees in late spring and summer [8,20] whereas individuals previously collected on herbaceous plants, within and outside the olive orchard, did not harbor the bacterium [20]. Additionally, nymphs have generally low mobility [22] and although they are able to acquire the pathogen, after molting they lose it, also losing the transmission capability [1]. Thus, it is suggested that major efforts should be made to control the vector through the suppression of nymphs on herbaceous hosts in early spring [8,9,20]. However, herb ground-covers can provide key requisites for important natural enemies of different olive pests [30,31], and improve the soil quality and prevent erosion [32,33]. Therefore, determining the plant species which host and do not host potential vectors of *X. fastidiosa* is essential to define a plant species composition of ground-cover vegetation that is able to support natural enemies of olive pests without favoring the presence of vectors.

The polyphagy of *P. spumarius* and *N. campestris* is well known [9,11,12,13], and *P. spumarius* potentially survives from moist to relatively dry environments, as long as the host plants are actively growing and not subjected to severe water stress [11]. However, due to the wide area of distribution of this species, the requirements for humidity could depend on the different ecotypes or biotypes [11]. Few studies have thoroughly analyzed the food resources of these species in Europe [3,9,12,13]. Therefore, it is urgent to understand the food resource preferences of vectors in Portugal, where *X. fastidiosa* has already been found and has many susceptible regions, such as the northeast of Portugal (high/medium risk of infection) [34].

To provide the knowledge required to establish protection and prevention measures against *X. fastidiosa* we aimed to: (i) identify preferred plant host species by nymphs; (ii) characterize the pattern of nymph aggregation within the foam; (iii) identify the preferred part of the plant where they feed (base, middle, or apical); and (iv) determine the period of nymph development of Aphrophoridae in olive orchards from the northeast of Portugal.

## 2. Material and Methods

### 2.1. Study Sites

The study was carried out in two olive orchards (a productive and an unproductive orchard) in northeastern Portugal (Mirandela) (Productive—P: 41°29′15.77″ N, 7°07′52.11″ W; Unproductive—UNP: 41°29′217.88″ N, 7°07′35.21″ W) under integrated production management and where potential vectors of *X. fastidiosa* were previously found [12]. The orchard P was in full production and the orchard UNP was hit by a strong fire in 2016, becoming unproductive and abandoned thereafter. The UNP orchard was selected because abandoned olive orchards may represent reservoirs of *X. fastidiosa* vectors. Both olive orchards had sizes of about 3.0 ha and spontaneous ground-cover that was mown after the ground vegetation dried at the beginning of the summer. The distance between trees varied from 7 to 9 meters and the age of trees varied from 18 to 80 years.

### 2.2. Experimental Design

From April to May 2017, from March to August 2018, and from March to June 2019 the plant species in the spontaneous ground-cover within the orchards were identified on a weekly basis (2017 and 2018) and each 10 days (2019). From March to May studies of nymphs were carried out. After May, identification of plants was carried out to record potential alternative herb/food sources in a context of environmental changes that may result in variations of the nymph development period. Samples and records of spittlebug nymphs and host/non-host plants were collected following the methodology described in [3,12,13]. Thus, thirty random rectangles (100 × 25 cm) were laid out in a diagonal transept of 100 m, covering around 1.0 ha. In each sample unit, the following data were recorded: (i) plant species with foams (hosts) and without foams (non-hosts); (ii) percentage of ground-cover by each plant species; (iii) number of Aphrophoridae foams per infested plant; (iv) location of the foam on the host plant (basal—2 cm from the ground; middle or apical—2 cm from the top of the plant); and (v) number of nymphs per foam (these were recorded but not collected in a non-interfering sampling). In 2018 and 2019 the species of Aphrophoridae were visually identified and development stages of nymphs in each sample unit were also recorded. Castroviejo [35] and Aizpuru et al. [36] were used for plant identification. Nymphs were identified in the field using Vilbaste [37]. In this work, *Neophilaenus* sp. species was not identified. However, we based the identification on a collection reference of adults from the same region and hosted at Polytechnic Institute of Bragança.

### 2.3. Data Analysis

To analyze the host preference, a linear mixed model (LMM) was used for each location and date. The response variable was the weighted number of nymphs per plant cover (w) (Equation (1)):

(1) $$w_{i}=\frac{plant\;cover_{i}}{100}\times \;nymphs\;number_{i}$$

where $w_{i}$ is the weighted number of nymphs (nymphs number) per the percentage of land covered (plant cover) for the plant i. The host plant was the explanatory variable, leveled by the identified taxa (species, genus or family). Random effects were the 30 sampled random rectangles. The models were validated by plotting residuals versus fitted values to assess the absence of patterns in the residuals. When residuals showed heterogeneity, data were log-transformed. The “lmer” function from the “lme4” package [38] in R [39] was used. For model results visualization “ggcoefstats” from “ggstatsplot” was used [40].

Statistical analysis was only possible for the data collected on 21 April and 26 April 2017 in the abandoned olive orchard, due to the low number of nymphs in the productive orchard and on the remaining dates.

## 3. Results

The most abundant Aphrophoridae was *P. spumarius*, followed by *Neophilaenus* sp. No further taxa were found. Only the genus was identified for *Neophilaenus* specimens, however adults in the reference collection, hosted at Polytechnic Institute of Bragança, mostly belong to *N. campestris*.

### 3.1. Plant Preference by Aphrophoridae Nymphs

**Figure 1:**
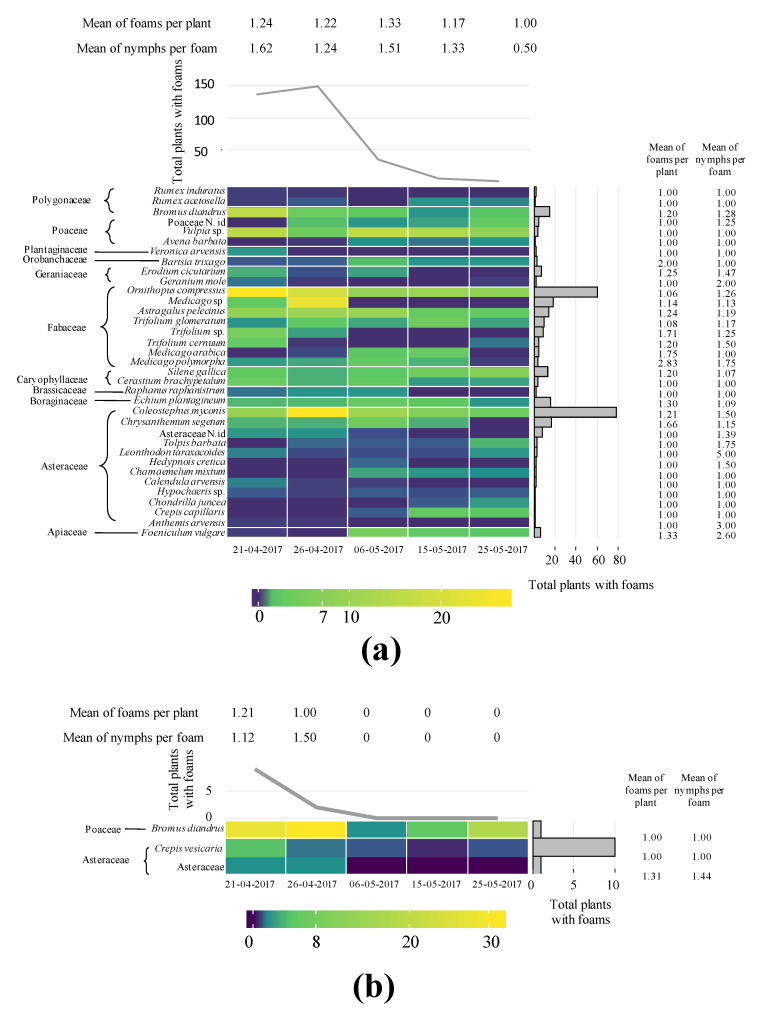
Heatmap plot showing 2017 data: (**i**) the percentage of plants with foam of all plants within a taxon (dark purple to yellow) (only host plants shown); (**ii**) total plants with foams during the collection period (top line graph); (**iii**) total plants with Aphrophoridae foams in the identified plants (right bar graph); (**iv**) mean of Aphrophoridae foams per infested plant and date; (**v**) mean of Aphrophoridae nymphs per foam per plant and date. (**a**) Integrated olive orchard burned in 2016 (UNP). (**b**) Productive integrated olive orchard (P). N. id. after taxa means not identified specimens belonging to that group. Families are indicated at the left of the plant species.

**Figure 2:**
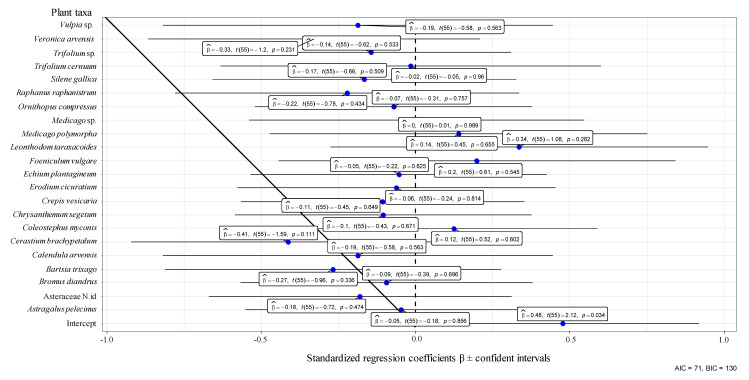
Estimated regression coefficients (β), t values (degrees of freedom), and *p*-values (within boxes) for the weighted number of nymphs by the plant cover as a function of each taxa identity in an abandoned olive orchard on 21 April 2017, obtained with linear mixed models. Blue points represent β and bars the confidence intervals. The dotted vertical line indicates the 0 value for the standardized regression coefficients. AIC: Akaike information criterion. BIC: Bayesian information criterion.

**Figure 3:**
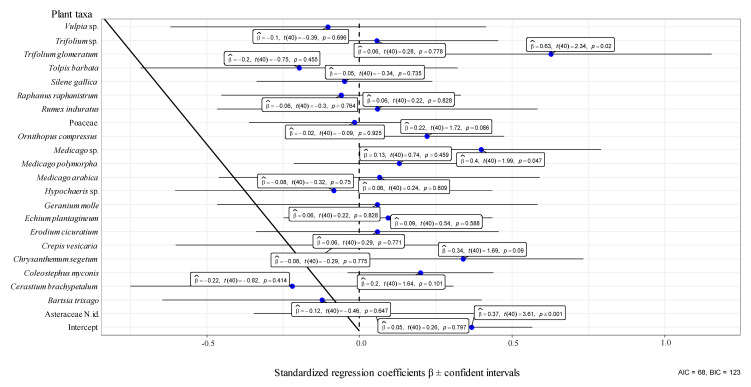
Estimated regression coefficients (β), t values (degrees of freedom), and *p*-values (within boxes) for the weighted number of nymphs by the plant cover as a function of each taxa identity in an abandoned olive orchard on 26 April 2017, obtained with linear mixed models. Blue points represent β and bars the confidence intervals. N. id. after taxa means not identified specimens belonging to that group. The dotted vertical line indicates the 0 value for the standardized regression coefficients. AIC: Akaike information criterion. BIC: Bayesian information criterion.

In the orchard UNP from 21 April to 25 May 2017, one year after the fire, a total of 66 plant taxa (species, genera, or families) belonging to 16 families were identified. The most abundant families were Asteraceae, Fabaceae, Poaceae, and Caryophyllaceae. Thirty-five taxa belonging to 11 families presented Aphrophoridae foams (Figure 1) and 33 belonging to 13 families did not (Figure S1). Generally, Aphrophoridae colonized the most abundant plants (Figure 1), whereas plants covering less than 2% of the ground were not colonized (Figure S1). The most colonized plant families were Asteraceae and Fabaceae. The plant species with most foams were *Coleostephus myconis* (L.) Cass (Asteraceae) and *Ornithopus compressus* L. (Fabaceae). Those plants corresponded with the most abundant species in the orchard during the development of Aphrophoridae nymphs. The nymphs presented a peak with 150 colonized plants at the end of April and decreased progressively until the end of May, with a total of 522 nymphs during the whole period (Figure 1). Other abundant plants, such as *Bromus diandrus* Roth (Poaceae), *Astragalus pelecinus* (L.) Barneby (Fabaceae), and *Chrysanthemum segetum* L. (Asteraceae), presented a low number of colonized plants. Caryophyllaceae and Brassicaceae species were colonized in low numbers. Generally, the LMM indicated that the consumed plants were not selected (Figure 2 and Figure 3), however on 26 April 2017 Asteraceae, *Medicago* sp. and *Trifolium glomeratum* L. were more colonized than expected (Figure 3). The mean number of foams was between one and three per plant species, and between one and two during the period of study. The mean number of nymphs per foam varied between one and five whereas the number of foams per infested plant varied between one and two throughout the period of study (Figure 1).

In the orchard P from 21 April to 25 May 2017 a total of 72 taxa (species, genera, or families) belonging to 17 families were identified, with Asteraceae, Fabaceae, and Poaceae the most abundant. The foam density was lower in the productive than in the non-productive olive orchard with a total of 23 nymphs during the whole period (Figure 1). *Crepis vesicaria* L., other not identified Asteraceae, and *B. diandrus* (Poaceae) presented Aphrophoridae foams in low numbers. Other plants did not present foams although some of them, such as *O. compressus*—Fabaceae or *Leonthodon taraxacoides* (Vill.) Mérat. and *C. myconis*–Asteraceae, covered more than 20% of the ground (Figure S2). The mean number of foams per infested plant and nymphs per foam throughout the period of study and per plant species was between one and two (Figure 1).

**Figure 4:**
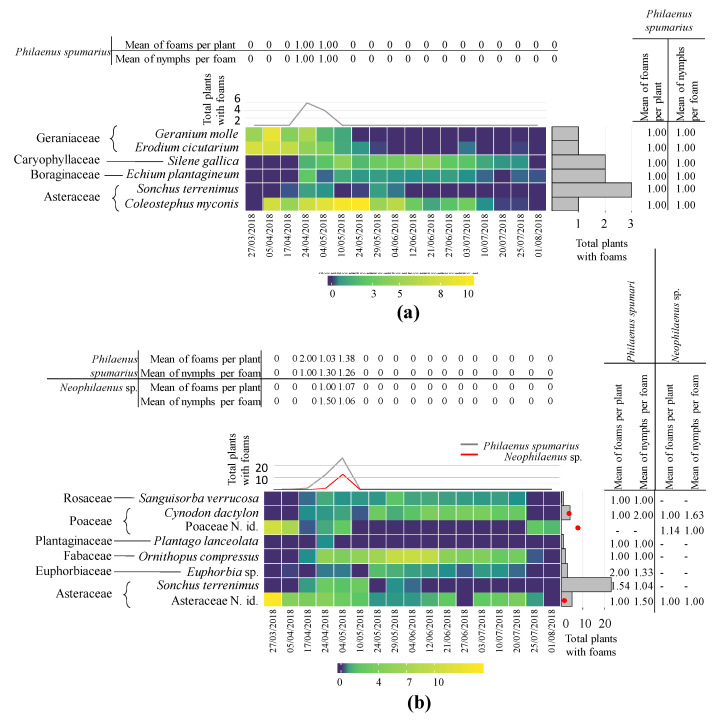
Heatmap plot showing 2018 data: (**i**) the percentage of plants with foam of all plants within a taxon (dark purple to yellow) (only host plants shown); (**ii**) total plants with foams of *Philaenus spumarius* (grey line) and *Neophilaenus* sp. (red line) during the collection period (top graphs); (**iii**) the total plants with *P. spumarius* (bars) and *Neophilaenus* sp. (points) foams in the identified plants (right graph); (**iv**) mean foams of *P. spumarius* and *Neophilaenus* sp. per plant and date; and (**v**) mean nymphs per foam of *P. spumarius* and *Neophilaenus* sp. per plant and date. (**a**) Abandoned olive orchard (UNP). (**b**) Productive integrated olive orchard (UP) N. id after taxa means not identified specimens belonging to that group. Families are indicated at the left of the plant species.

In the orchard UNP from 21 March to 1 August 2018, two years after the fire, a total of 116 taxa (species, genera, or families) belonging to 24 families were identified. The most abundant families were Asteraceae, Fabaceae, and Poaceae. Six plant species (four families) were colonized by Aphrophoridae, all belonging to *P. spumarius* nymphs. Asteraceae, represented by *Sonchus terrenimus* L. and *C. myconis*, was the most colonized family. The number of total plants with foams varied between one and three per plant species, and the peak of total recorded foams was six at the beginning of May (Figure 4). The mean number of foams per infested plant and nymphs per foam in the plant species and during the period of study was one (Figure 4). Abundant plant species during the development of *P. spumarius* nymphs, such as *O. compressus*, *Trifolium* spp. (Fabaceae), *C. segetum* (Asteraceae), or *Bromus* spp. (Poaceae), were not colonized by Aphrophoridae nymphs (Figure S3). A relevant number of rare species (<1% of coverage during the period of study) did not present Aphrophoridae nymphs (Figure S4).

In the orchard P from 21 March to 1 August 2018, a total of 125 taxa (species, genera, or families) belonging to 26 families were identified. The most abundant families were Asteraceae, Poaceae, and Fabaceae. A total of eight taxa presented Aphrophoridae. The Asteraceae *S. terrenimus* and other not identified Asteraceae presented a higher number of *P. spumarius* nymphs, whereas *Neophilaenus* sp. was more abundant on *Cynodon dactylon* (L.) Pers. and other Poaceae. The peak of both Aphrophoridae species was at the beginning of May, with *P. spumarius* being more abundant (25) than *Neophilaenus* sp. (11). The mean number of foams per infested plant and nymphs per foam in the plant species and during the period of study varied between one and two (Figure 4). Abundant plant species that occurred during Aphrophoridae nymphal development, such as *C. myconis* or *Hypochoeris* sp. (Asteraceae), *A. pelecinus* or *Hymenocarpos lotoides* (L.) Vis. (Fabaceae)*, Molineriella laevis* (Brot.) Rouy, or *Bromus* spp. (Poaceae) did not present any foams (Figure S5). A relevant number of rare plant species (<1% of coverage during the period of study) did not present any Aphrophoridae nymphs (Figure S6).

In the orchard UNP from 22 March to 14 June 2019, three years after the fire, a total of 80 taxa (species, genera, or families) belonging to 22 families were identified. The most abundant families were Asteraceae, Fabaceae, and Poaceae, which were represented by abundant plant species such as *C. myconis* and *Hypochaeris glabra* L. (Asteraceae), *O. compressus*, *Trifolium* spp. and *A. pelecinus* (Fabaceae), *Bromus* spp., *C. dactylon*, and *Vulpia* sp. (Poaceae) (Figure S7). No foams were observed during 2019 in the orchard UNP. A relevant number of rare species (<1% of coverage during the period of study) did not present any Aphrophoridae nymphs (Figure S8).

**Figure 5:**
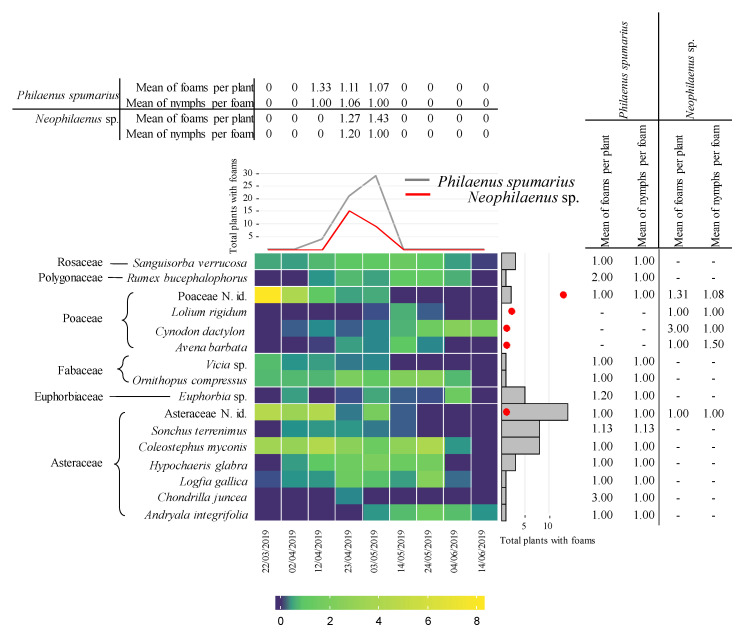
Heatmap plot showing 2019 data: (**i**) the percentage of plants with foam of all plants within a taxon (dark purple to yellow) (only host plants shown); (**ii**) total plants with foams of *Philaenus spumarius* (grey line) and *Neophilaenus* sp. (red line) during the collection period (top graphs); (**iii**) the total plants with *P. spumarius* (bars) and *Neophilaenus* sp. (points) foams in the identified plants (right graph); (**iv**) mean foams of *P. spumarius* and *Neophilaenus* sp. per plant and date; and (**v**) mean nymphs per foam of *P. spumarius* and *Neophilaenus* sp. per plant and date in a productive integrated olive orchard (P). N. id after taxa means not identified specimens belonging to that group. Families are indicated at the left of the plant species.

In 2019 in the orchard P a total of 77 taxa (species, genera, or families) belonging to 23 families were identified. The most abundant families were Asteraceae, Fabaceae, and Poaceae. Ten plant species or genera (six families) were colonized by *P. spumarius* nymphs. Asteraceae, represented by *S. terrenimus*, *C. myconis*, *Chondrilla juncea* L., *Andryala integrifolia* L., *H. glabra*, and *Logfia gallica* (L.) Coss. & Germ., was the most colonized family. Four plant species (Poaceae) and few not identified Asteraceae were colonized by *Neophilaenus* sp. The number of total plants with foams varied between one and 14 per plant species. The peak of total recorded foams was 29 at the beginning of May and 15 at the end of April for *P. spumarius* and *Neophilaenus* sp., respectively (Figure 5). The mean of foams per infested plant and nymphs per foam in the plant species and throughout the period of study was between one and three for both species (Figure 5). Abundant plant species during the development of both species nymphs, such as *O. compressus*, *Trifolium* spp. (Fabaceae), *C. segetum* (Asteraceae), or *Bromus* spp. (Poaceae), did not present any foam (Figure S9). A relevant number of rare species (<1% of coverage during the period of study) did not present any Aphrophoridae nymphs (Figure S10).

### 3.2. Distribution of Foams Along Plant Stems

**Figure 6:**
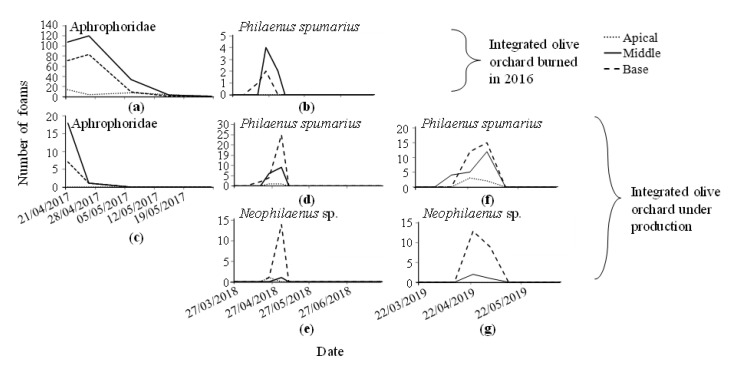
Number of Aphrophoridae foams in the apical, middle, or base part of the host plant in an abandoned olive orchard (UNP) during the sampled dates: (**a**) Aphrophoridae, year 2017 and (**b**) *Philaenus spumarius*, year 2018; and a productive olive orchard (P): (**c**) Aphrophoridae, year 2017, (**d**) *P. spumarius*, year 2018, (**e**) *Neophilaenus* sp., year 2019, (**f**) *P. spumarius*, year 2018, (**g**) *Neophilaenus* sp., year 2019.

**Figure 7:**
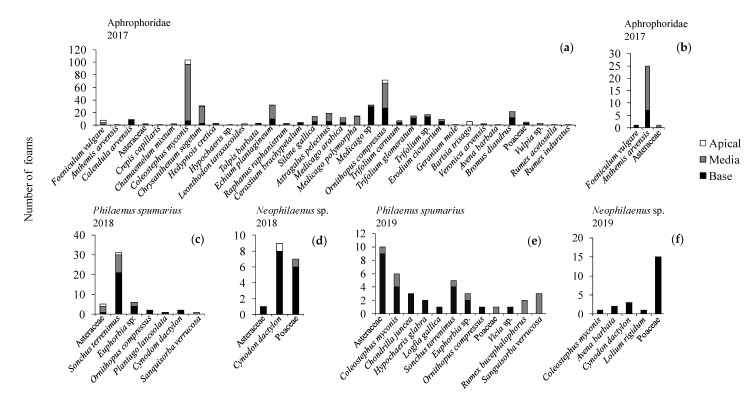
Number of given nymphal foams in the apical, middle, or base part of the host plant in: (**a**) an abandoned olive grove (UNP)—Aphrophoridae, 2017; (**b**) an integrated olive grove (P)—Aphrophoridae, 2017; (**c**) P—*Philaenus spumarius*, 2018; (**d**) P—*Neophilaenus* sp., 2018; (**e**) P—*P. spumarius*, 2019; (**f**) P—*Neophilaenus* sp., 2019.

In 2017, when Aphrophoridae were more abundant, most of the foams were found in the middle part of the plant, followed by the base. Few were located in the apical part of the plant. In 2018 and 2019, the abundance of foams was too low to show a pattern, however, generally more foams were observed at the base of the plant (Figure 6 and Figure 7).

### 3.3. Dynamics of Nymphal Instars

**Figure 8:**
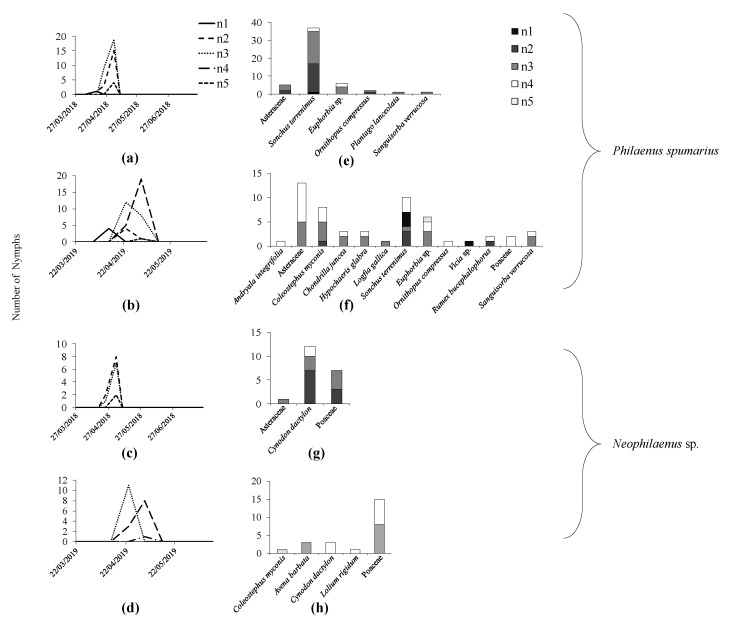
Number of *Philaenus spumarius* (**a**,**b**,**e**,**f**) and *Neophilaenus* sp. (**c**,**d**,**g**,**h**) nymphs in different developmental stages (n1, n2, n3, n4, n5) in a productive olive orchard (P) during the sampled dates (2018 and 2019) (**a**–**d**) and on the host plants (**e**–**h**).

The development periods of the different nymph stages during 2018 and 2019 are shown in Figure 8. Overall, there was no clear preference of a given nymphal stage of *P. spumarius* or *Neophilaenus* for a specific host plant (Figure 8).

## 4. Discussion

In this study, the host/non-host plants of Aphrophoridae nymphs in the herbaceous ground-cover, the distribution along the plant stems, and the period of nymph development were identified in olive orchards from the northeast of Portugal, an important olive growing region and one that is susceptible to *X. fastidiosa* epidemics.

The most abundant Aphrophoridae was *P. spumarius* followed by *Neophilaenus* sp., which is in agreement with the observations in other Mediterranean countries [3,9,12,13,15]. Nevertheless, no other species commonly found, such as *Aphrophora* sp. in Italy [3,13,15] or *Lepyronia coleoptrata* (L.) in Spain [12], were identified in northeastern Portugal. The number of individuals reported in Italy [3,9,13,15] was by substantially higher than that observed in the present study and in some Spanish regions [12]. However, even low densities of transmitting individuals could account for considerable incidence of *X. fastidiosa* diseases over the years [12,41]. In addition, the hatching and nymph development period of Aphrophoridae showed differences among locations [3,9,13,14,15]. These variations have been associated with a non-linear component in the temperature-dependent development rate function [3,15].

The polyphagy of *P. spumarius* is well known [11,13,14,22] and makes it difficult to establish the plant diversity for ground-cover vegetation in olive orchards. In this study, the most colonized species belong to the families Asteraceae and Fabaceae, and few Apiaceae species were colonized. Other field studies found that species of these three families were highly colonized [3,9,12,13], with the exception of Bodino et al. [13], who also found a low colonization of Apiaceae species. In the northeast of Portugal, a delay of Apiaceae development with respect to Aphrophoridae nymphs could result in low colonization. However, adults were observed feeding on *F. vulgare* (personal observation). Generally, no preference was found among the colonized plants. Some abundant plants, such as *B. diandrus* (Poaceae), *C. segetum* (Asteraceae), *A. pelecinus,* several species of *Trifolium* sp. (both Fabaceae), and species of Caryophyllaceae and Brassicaceae, were barely colonized (which makes them interesting for selective grassing). Although not identified Asteraceae, *Medicago* sp., and *T. glomeratum* (both Fabaceae) were more colonized than expected at the end of April in an olive orchard abandoned after a fire. Importantly, the high abundance in the surrounding environment of a host plant could mask a potential preference by decreasing the value of the selection measure, which could be positive with lower abundances of the plant. For example, Bodino et al. [13] found *Medicago* sp. to be the plant genus with more *P. spumarius* nymphs. In their case, *Medicago* was not positively selected because it was also one of the most abundant plants in one of the regions, but the selection index was positive in another region where it was less abundant (similarly to our results). Thus, for better understanding whether *P. spumarius* colonizes the occurring herbs regardless of their identity, or otherwise prefers specific plant species, further research should analyze the preference of *P. spumarius* under controlled abundance of plants (comparing for example a potential important host plant, such as *Medicago* sp., vs. an apparently less colonized Fabaceae, such as *A. pelecinus*, in similar abundance conditions). Moreover, although we did not find avoided plants in other regions, several species (e.g., some Poaceae, *Oxalis* (Oxalidaceae), *Lysimachia* (Myrsinaceae), *Geranium* (Geraniaceae), *Papaver* (Papaveraceae), *Fumaria* (Fumariaceae), and *Raphanus* (Brassicaceae)) were found to be avoided by *P. spumarius* nymphs [9] or negatively selected (*Hyoseris* sp.) [13]. To obtain reliable recommendations, further research is needed to analyze the effect of vegetal ground-covers with different plant composition on *X. fastidiosa* vectors, olive pests, and their natural enemies, in addition to erosion and soil fertility.

In the present study the mean of Aphrophoridae nymphs per foam varied between 1 and 3 (only one plant of *L. taraxacoides* presented one foam with five nymphs). However, typically, nymphs of Aphrophoridae feed gregariously within a foam [42]. Wise et al. [42] suggested that the optimal group size of spittlebug nymphs depends on a compromise between bottom-up and within-trophic-level influences. Bierdermann [43] found that aggregation of nymphs inside the foams was responsible for a reduction of mortality in all instars for *Neophilaenus albipennis* (F.). This species passively aggregated up to four nymphs per spittle and showed a reduction of the aggregation in bigger instars. Further studies are needed to elucidate factors triggering the aggregation pattern in the region and its influence on nymph survival.

Regarding the distribution of foams along the plant stems, we found more foams in the middle than in the apical and basal parts of the plants. Similarly, Grant et al. [44] found most *P. spumarius* nymphs on the leaf internodes along the main stem of *Carduus nutans* L. but few were found in the rosettes. However, our observations are mainly from late April 2017 (the only dates with sufficient nymphs) and nymphs may shift their feeding preferences with the progress of the season; as previously found by Bodino et al. [13], nymphs shift from rosettes to axil leaves as the base part hardens with the season progress, attempting to maintain the foam size and hydration, in addition to the feeding on soft plant parts. Similarly, some authors found that plants providing wide leaf axils and protected feeding sites were attractive for *P. spumarius* nymphs [13,45]. This is in agreement with the morphology of some colonized plants by *P. spumarius* in the present study (e.g., *C. myconis*-lobate leaves and *S. terrenimus*-pinnatisct leaves including large leaflets) but not others (e.g., *O. compressus* pinnatisect leaves with small leaflets). Other factors, such as the sample hour in the day or the nymph development, could have an effect; for example, according to Weaver et al. [14], early in the morning foams may be found in the top of the plants, but as the temperature rises, the foams dry up and nymphs move down to the lower part of the stem. Hoffman and McEvoy [46] also found that later nymphal instars can probably feed on a wider range of plants.

The results of our work indicated that in northeastern Portugal nymph development begins in early April and ends in early May when they molt to the adult stage. Bodino et al. [15] suggest that measures for controlling the whole nymphal population would achieve the maximum efficacy targeting the 4th instar nymphs. Therefore, the end of April (before the adult emergence) would most likely be the best period for controlling nymphs through ground-cover suppression, as is often recommended (see introduction section). However, in recent decades, the Common Agricultural Policy (CAP) has recommended and/or promoted the maintenance and implementation of cover crops for a sustainable agriculture [33,47]. The suppression of ground-cover—weeds and shrubs—could have some disadvantages such as: (i) hampering natural enemies by reducing other important resources (such as oviposition and overwintering places or alternative food resources, including pollen and nectar) for natural enemies of both vectors and other pests [30,31]; and (ii) damaging biological, chemical, and physical conditions of soils including fertility and nutrient storage, water storage, soil structure, and erosion [32,33]. In addition, natural enemies may have an impact on the control of vectors of *X. fastidiosa*, although these are little studied in Europe [48,49,50]. Among these, egg parasitoids may represent an important means of vector control. Several egg parasitoids (such as *Ooctonus vulgatus* Haliday (1833), *Gonatocerus* sp.—family Mymaridae, *Oligosita* sp.—family Trichogrammatidae) of *X. fastidiosa* vectors/potential vectors are described [3,50], and in the northeast of Portugal some preliminary results indicate the existence of egg parasitoids of *X. fastidiosa* vectors/potential vectors [51]. Therefore, an excessively early removal of the ground vegetation might damage this parasitoid population by eliminating the parasitized eggs before the parasitoids hatch. Additionally, parasitoids of eggs can be particularly important because the protection provided by the foam probably reduces the vector susceptibility to natural enemies during the nymphal stage [17,18]. Thus, an ideal strategy of vector control should maintain a vegetal ground-cover which does not favor nymphs but enhances the natural enemies of olive pests and *X. fastidiosa* vectors, in addition to protecting the soil against erosion.

A higher number of Aphrophoridae nymphs was observed in the abandoned olive orchard one year after the fire. This orchard presented an atypical dense and vigorous herbaceous ground-cover, which was likely derived from the higher availability of soil nutrients. In accordance, previous research [11,14] suggested that preference revealed in settled places and the production of the foam depends on humidity and water availability [11] and fertilization [52]. The plant consumption varied with the year and location, and some abundant plants, such as *C. myconis* or *O. compressus*, in some situations were colonized by the nymphs but not in others. This indicates that factors other than the taxonomic characteristics may influence the detection of targeted plants. In accordance, *Sherardia* sp. (Rubiaceae) was found to be negatively selected by Dongiovanni et al. [9] but preferred by Serio et al. [3]. Other studies also found variability in the richness of colonized plants in different years and locations [3,9,12,13]. Finally, the present study was not able to establish a pattern of preference by the nymph stages, probably due to insufficient numbers of nymphs, in agreement with Serio et al. [3]. However, Bodino et al. [13] recorded different plant preference among nymph stages. These aspects highlight the complexity of the plant selection by Aphrophoridae nymphs. Plant characteristics, such as the plant morphology, nutritional composition, water availability [9,53], and tactile cues, or characteristics of the spittlebugs, such as visual and vibrational detection skills or olfactory stimuli [54,55,56], could be involved.

In relation to *Neophilaenus* sp. nymphs, the Poaceae family was the most colonized, and the few individuals found were mainly at the base of the plants. This is in agreement with the findings in other regions for *N. campestris* [3,12,13] or *Neophilaenus* spp. [9], although Morente et al. [12] also found *N. campestris* on *Trifolium campestre* Schreb.

## 5. Conclusions

In northeastern Portugal the most abundant Aphrophoridae was *P. spumarius* followed by *Neophilaenus* sp. Aphrophoridae nymphs developed from April to early May with a peak in mid-April. Therefore, early April would be the optimal period for vector control. Moreover, nymphs showed a low aggregation behavior. *Philaenus spumarius* fed mostly on the Asteraceae *C. myconis* and *S. terrenimus*, and the Fabaceae *O. compressus* and *Neophilaenus* sp. on Poaceae. However, only Asteraceae (not identified), *Medicago* sp. and *T. glomeratum* presented more nymphs than expected in an abandoned olive orchard and only on one sampling date. Some species, such as *O. compressus*, seem to be highly consumed in some years and locations but not in others. It is likely that several factors other than taxonomic characteristics are involved in the plant selection by Aphrophoridae nymphs. Additionally, some abundant plants, such as *B. diandrus*, *A. pelecinus*, *C. segetum*, several species of *Trifolium* sp., and species of Caryophyllaceae and Brassicaceae, generally were barely or not colonized. These results establish the basis for further research about the design of vegetal ground-covers which enhance natural enemies of olive pests, reduce erosion, or promote soil fertility without favoring *X. fastidiosa* vectors, particularly in *Xylella*-free regions where lower-impact measures are required. However, given that Aphrophoridae show different behaviors in different situations, long-term research is necessary to identify patterns and optimal vegetal ground-covers. Our results suggest that in the region of study, the Aphrophoridae populations are generally low. Therefore, intense regional monitoring with particular attention to abandoned, post-fire, fertilized, and watered fields (where vector populations could increase), followed by ground-cover removal in areas with elevated Aphrophoridae populations, may prevent or retard the *X. fastidiosa* infection in the region. However, further research of the effect of ground-cover with different plant compositions is urgently needed to guarantee a sustainable agriculture.

## Supplementary Materials

The following are available online at https://www.mdpi.com/2075-4450/11/10/720/s1, Figure S1: Non-host plant of Aphrophoridae in an olive orchard abandoned after a fire (year 2017); Figure S2: Non-host plant of Aphrophoridae in an integrated olive orchard (year 2017); Figure S3: Non-host plant (≥1%) of Aphrophoridae in an olive orchard abandoned after a fire (year 2018); Figure S4: Non-host plant (<1%) of Aphrophoridae in an olive orchard abandoned after a fire (year 2018); Figure S5: Non-host plant (≥1%) of Aphrophoridae in an integrated olive orchard (year 2018). Figure S6: Non-host plant (<1%) of Aphrophoridae in an integrated olive orchard (year 2018). Figure S7: Non-host plant (≥1%) of Aphrophoridae in an olive orchard abandoned after a fire (year 2019). Figure S8: Non-host plant (<1%) of Aphrophoridae in an olive orchard abandoned after a fire (year 2019). Figure S9: Non-host plant (≥1%) of Aphrophoridae in an integrated olive orchard (year 2019). Figure S10: Non-host plant (<1%) of Aphrophoridae in an integrated olive orchard (year 2019).
