# Supplementary material for: Populations and Host/Non-Host Plants of Spittlebugs Nymphs in Olive Orchards from Northeastern Portugal
Source: Insects. 2020 Oct 21;11(10):720. doi: 10.3390/insects11100720 (PMC7589065; doi:10.3390/insects11100720)
Supplement: Supplementary file 1 [file insects-11-00720-s001.pdf]

Non-host plant of Aphrophoridae in an adandoned after a fire olive orchard (year 2017)

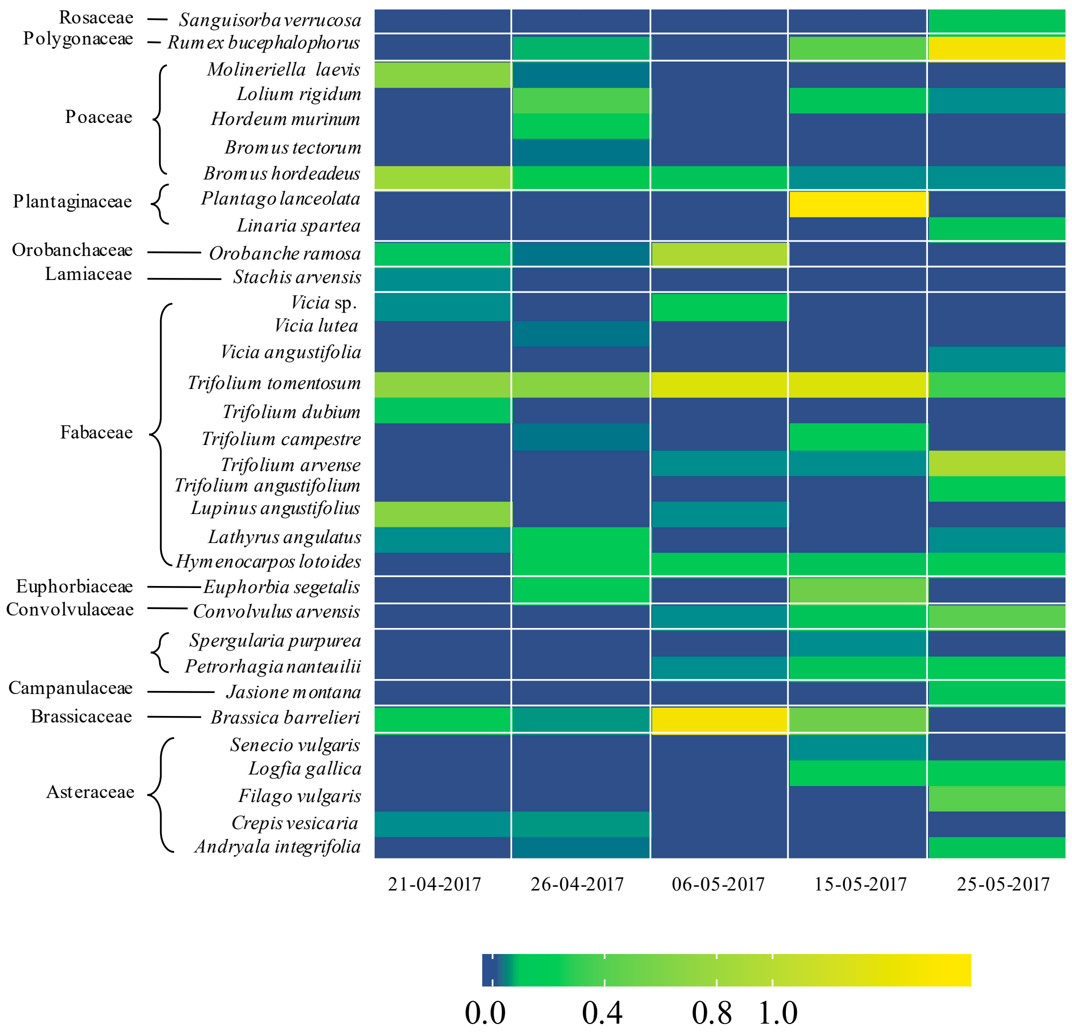

Figure S1. Heatmap plot showing the percentage of plant taxa identified through the collection period (year 2017) (non-host plants) (dark purple to yellow) in an abandoned after a fire olive orchard. Families are indicated at the left of the plant species name.

Non-host plant of Aphrophoridae in an integrated olive orchard (year 2017)

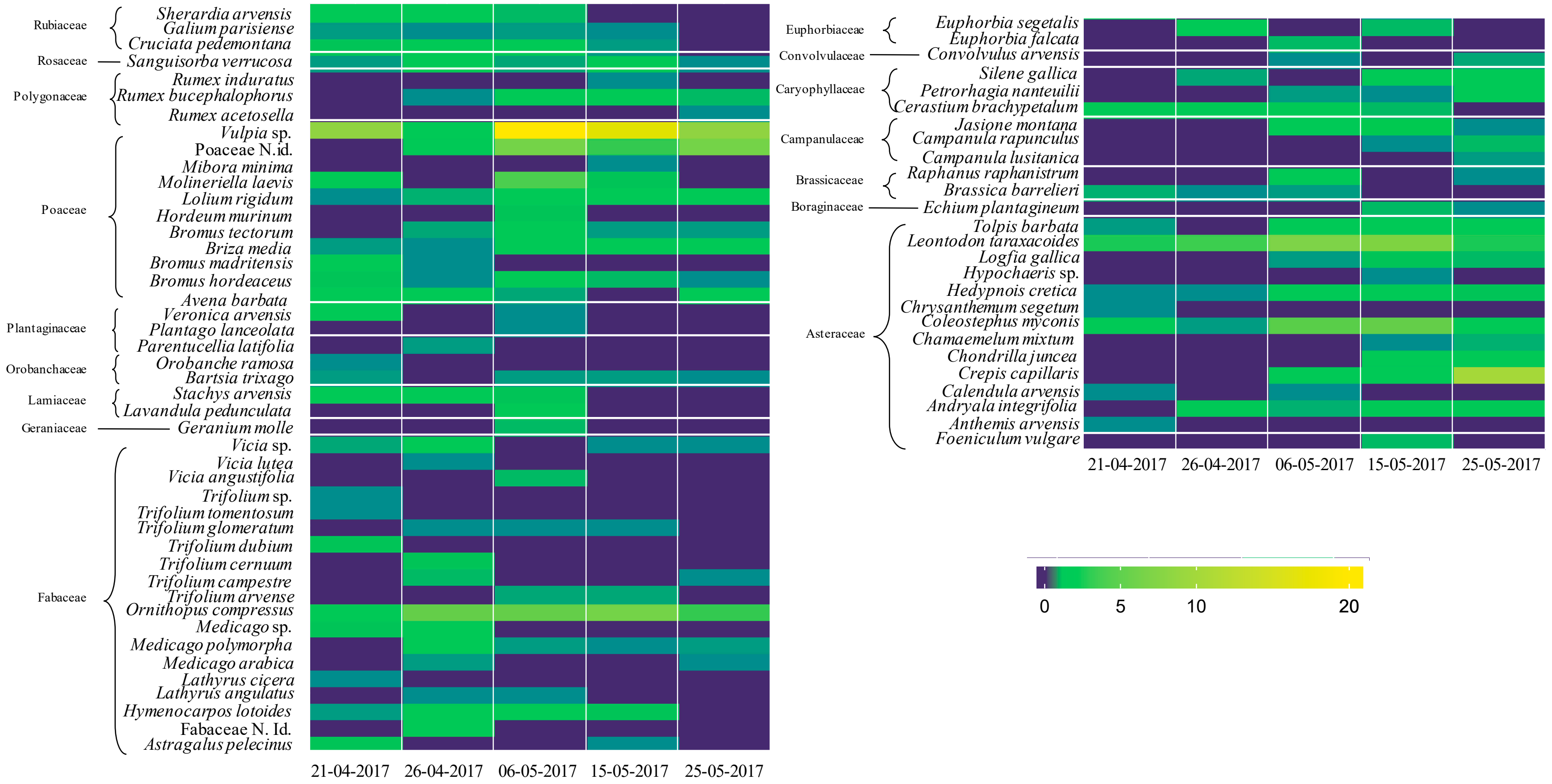

Figure S2. Heatmap plot showing the percentage of plant taxa identified through the collection period (year 2017) (non-host plants) (dark purple to yellow) in an integrated olive orchard. N. id after taxa means not identified specimens belonging to that group. Families are indicated at the left of the plant species name.

Non-host plant (≥1%) of Aphrophoridae in an abandoned after a fire olive orchard (year 2018)

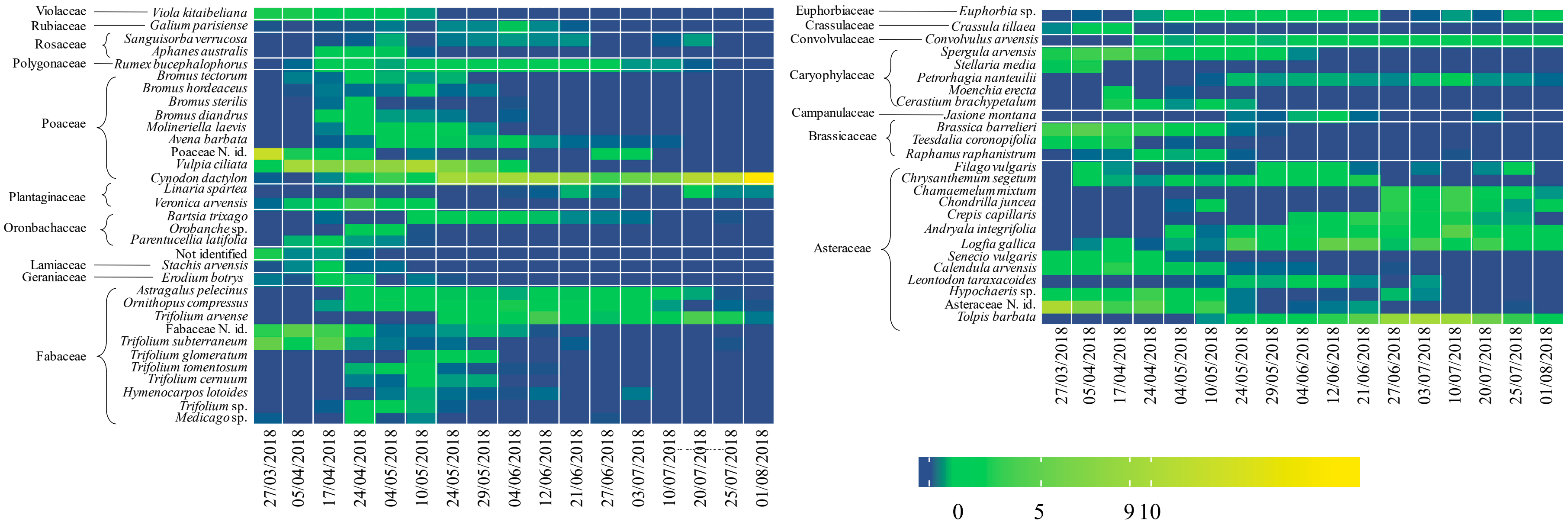

Figure S3. Heatmap plot showing the percentage of plant taxa identified with ≥ 1% of coverage through the collection period (year 2018) (dark purple to yellow) (non-host plants) in an abandoned after a fire olive orchard (non-host plants). N. id. after taxa means not identified specimens belonging to that group. Families are indicated at the left of the plant species name.

Non-host plant (<1%) of Aphrophoridae in an abandoned after a fire olive orchard (year 2018)

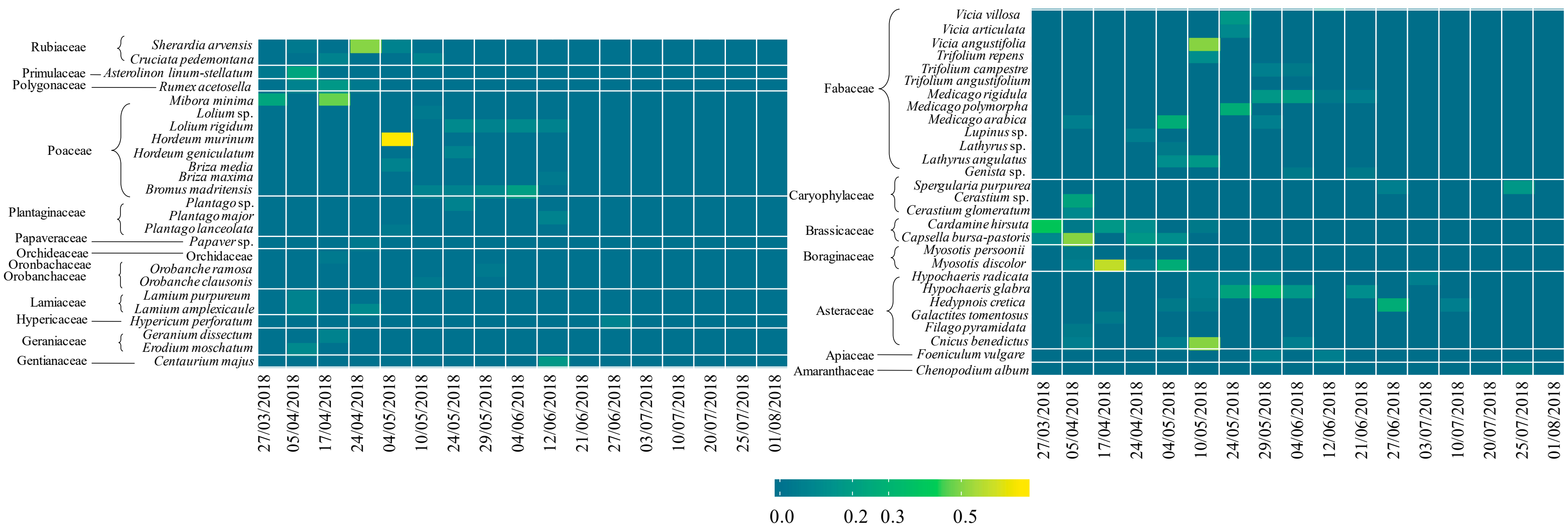

Figure S4. Heatmap plot showing the percentage of plant taxa identified with <1% of coverage through the collection period (year 2018) (dark purple to yellow) in an abandoned olive orchard (non-host plants). N. id after taxa means not identified specimens belonging to that group. Families are indicated at the left of the plant species name.

Non-host plant (≥1%) of Aphrophoridae in an integrated olive orchard (year 2018)

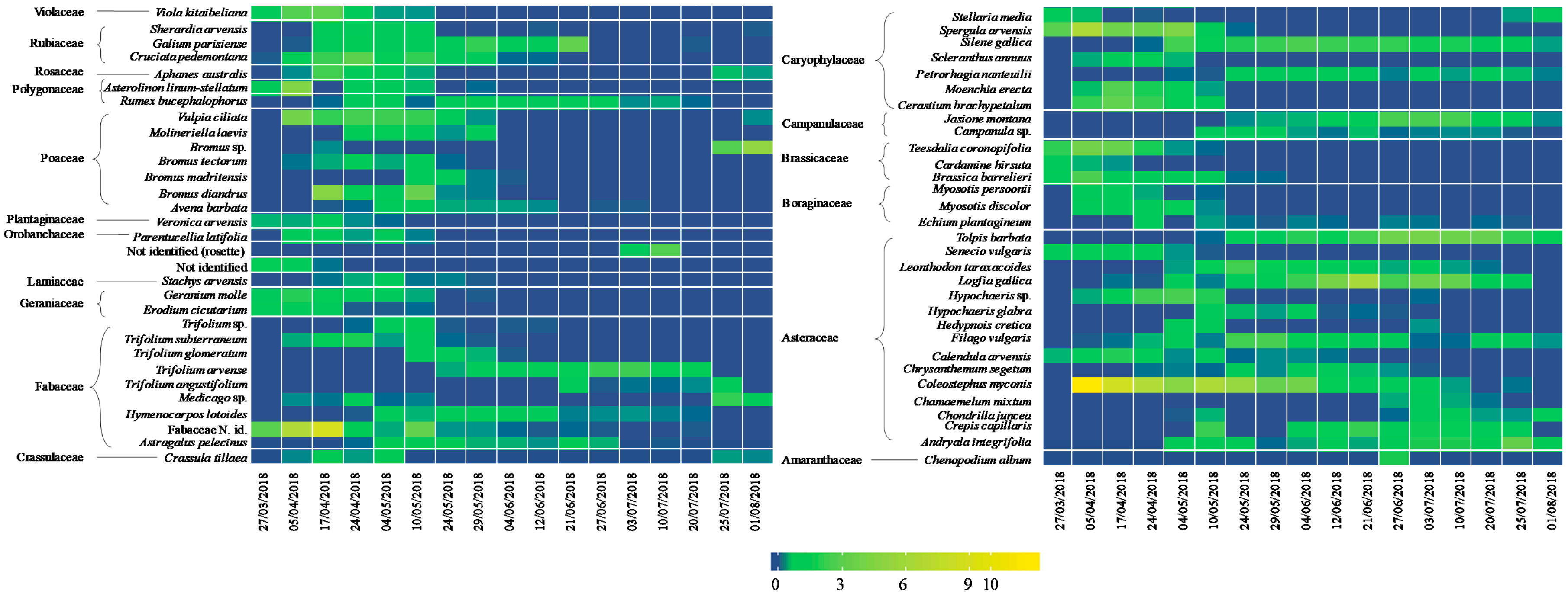

Figure S5. Heatmap plot showing the percentage of plants taxa identified with ≥1% of coverage through the collection period (year 2018) without Aphrophoridae nymphs. (dark purple to yellow) in an integrated olive orchard (non-host plants). N. id. after taxa means not identified specimens belonging to that group. Families are indicated at the left of the plant species name.

Non-host plant (<1%) of Aphrophoridae in an integrated olive orchard (year 2018)

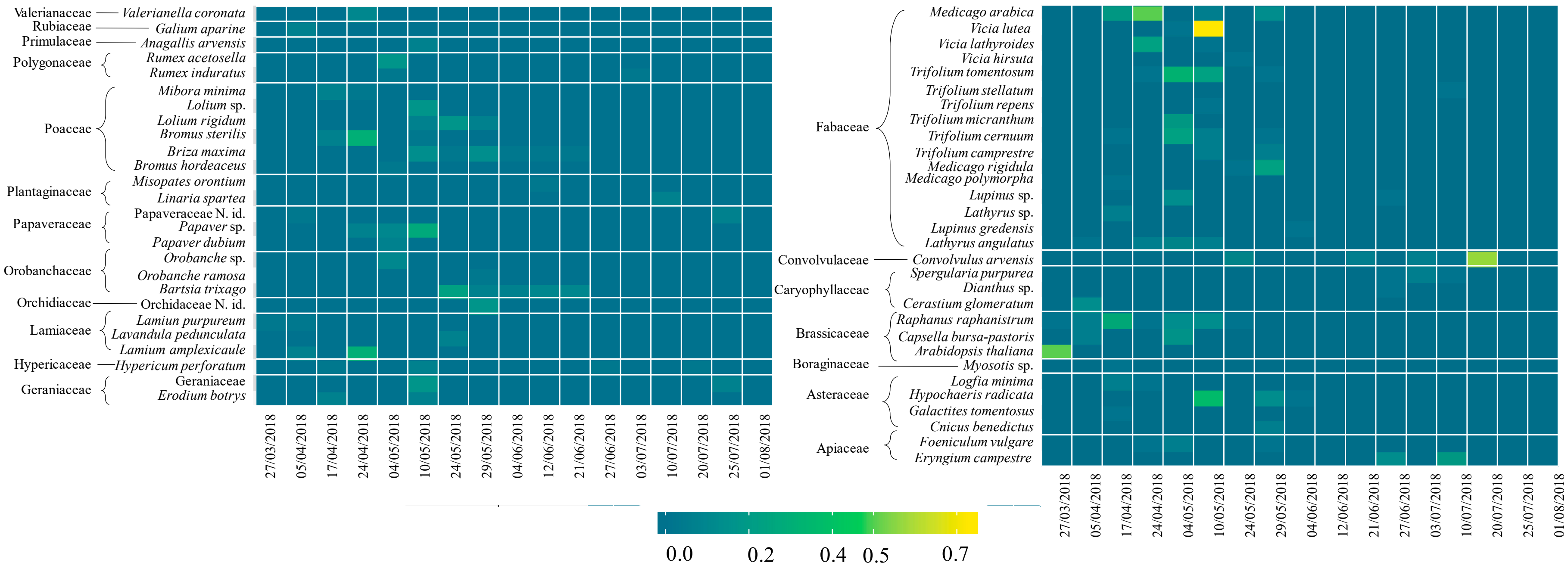

Figure S6. Heatmap plot showing the percentage of plant taxa identified with <1% of coverage through the collection period (year 2018) without Aphrophoridae nymphs (dark purple to yellow) in an integrated olive orchard (non-host plants). N. id. after taxa means not identified specimens belonging to that group. Families are indicated at the left of the plant species name.

Non-host plant (≥1%) of Aphrophoridae in an abandoned after a fire olive orchard (year 2019)

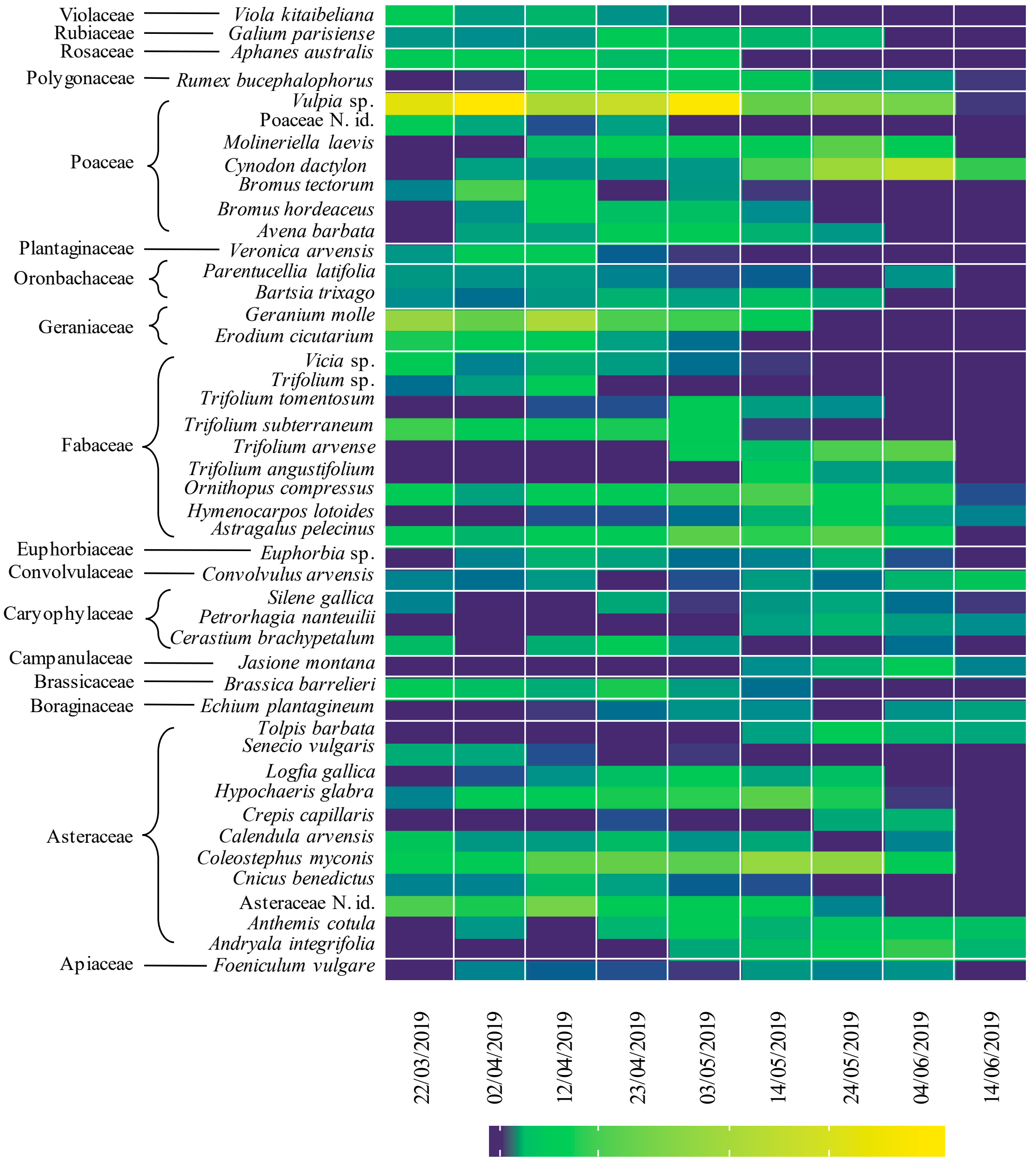

Figure S7. Heatmap plot showing the percentage of plant taxa identified with ≥ 1% of coverage through the collection period (year 2019) without Aphrophoridae nymphs (dark purple to yellow) in an abandoned olive orchard (non-host plants). N. id. after taxa means not identified specimens belonging to that group. Families are indicated at the left of the plant species name.

Non-host plant (<1%) of Aphrophoridae in an abandoned after a fire olive orchard (year 2019)

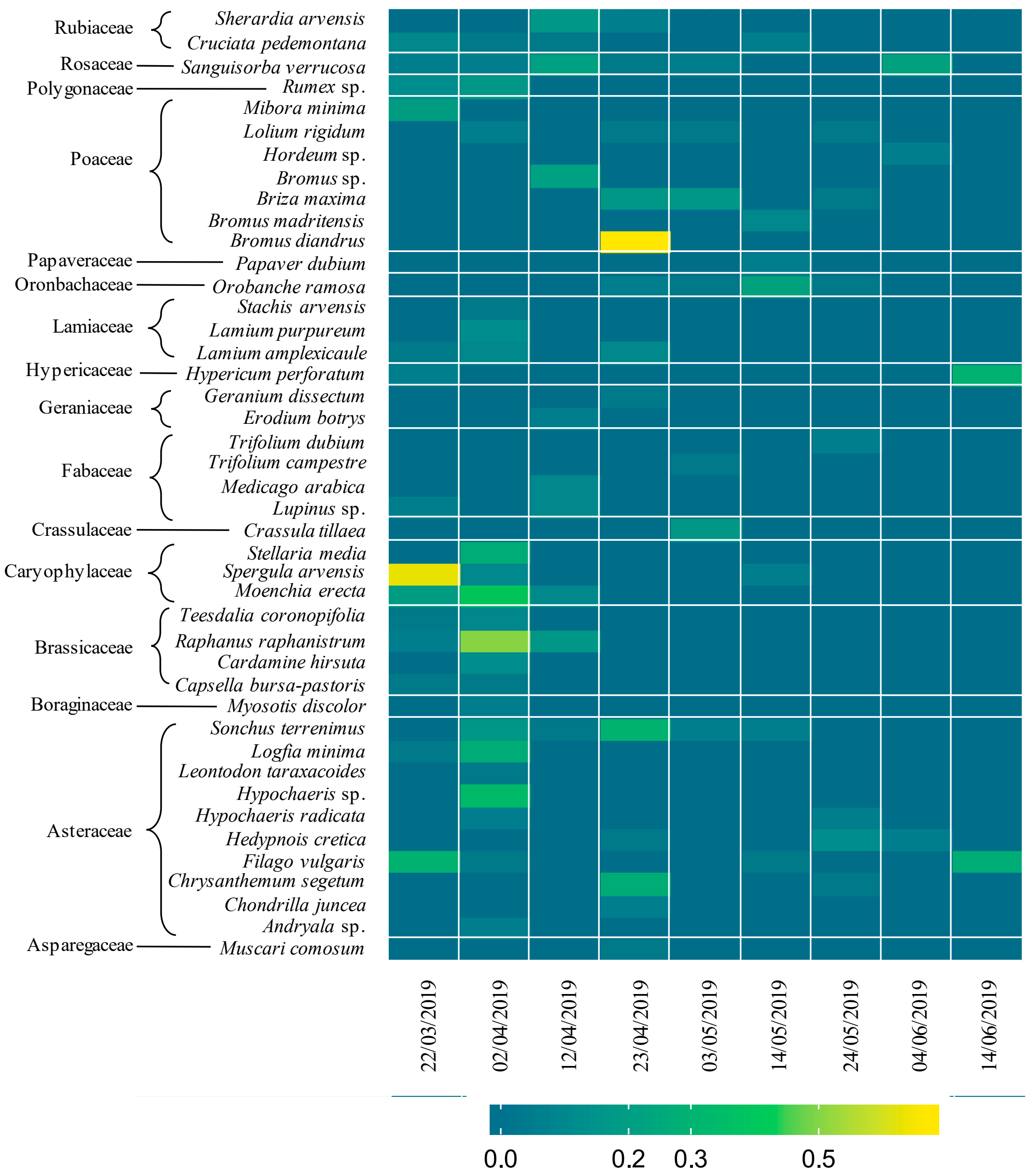

Figure S8. Heatmap plot showing the percentage of plant taxa identified with <1% of coverage through the collection period (year 2019) without Aphrophoridae nymphs (dark purple to yellow) in an abandoned after a fire olive orchard (non-host plants). Different colors at the right of the plant name indicate the plant family. Families are indicated at the left of the plant species name.

Non-host plant (≥1%) of Aphrophoridae in an integrated olive orchard (year 2019)

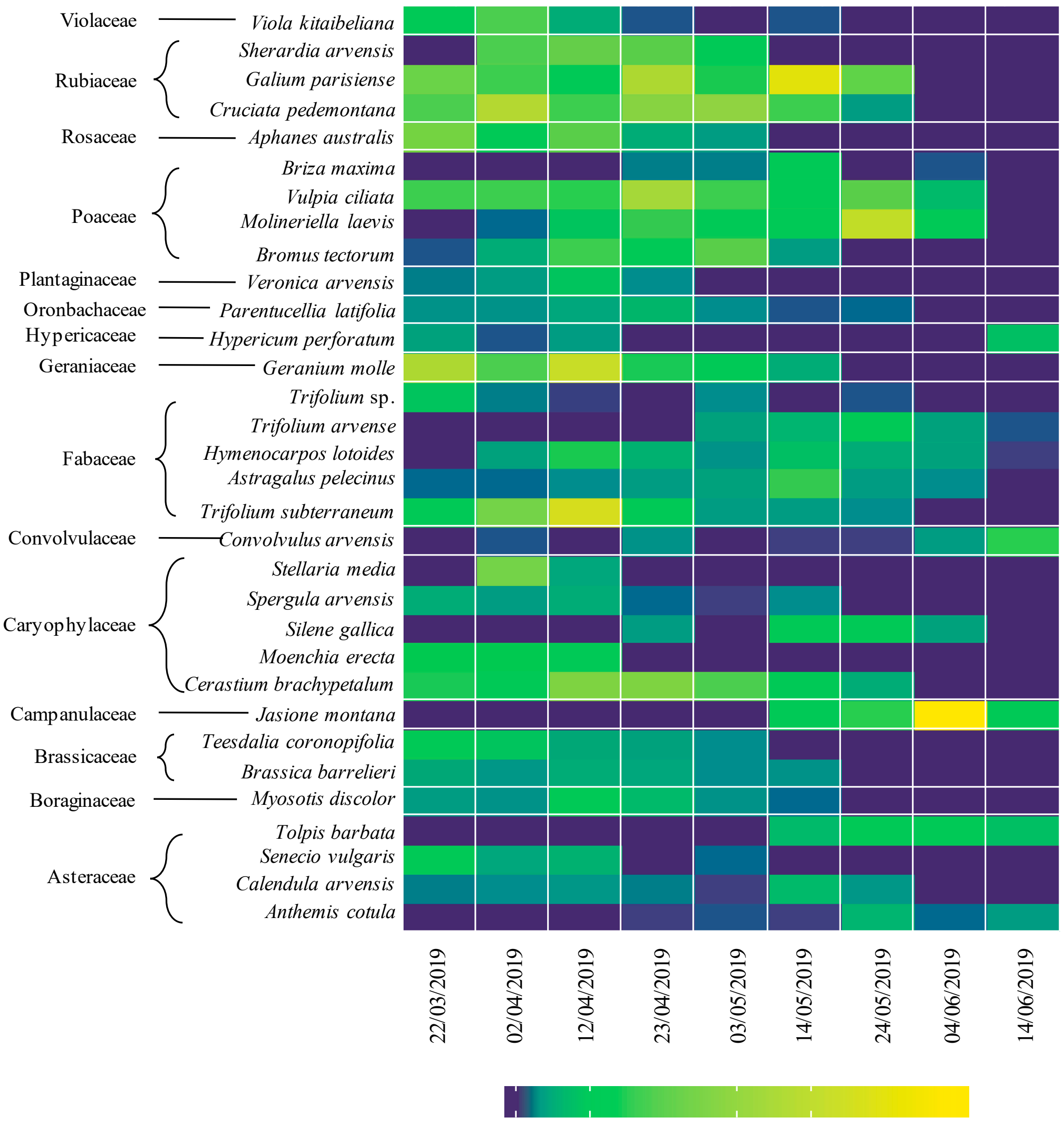

Figure S9. Heatmap plot showing the percentage of plant taxa identified with ≥ 1% of coverage through the collection period (year 2019) without Aphrophoridae nymphs (dark purple to yellow) in an integrated olive orchard (non-host plants). Families are indicated at the left of the plant species name.

Non-host plant (<1%) of Aphrophoridae in an integrated olive orchard (year 2019)

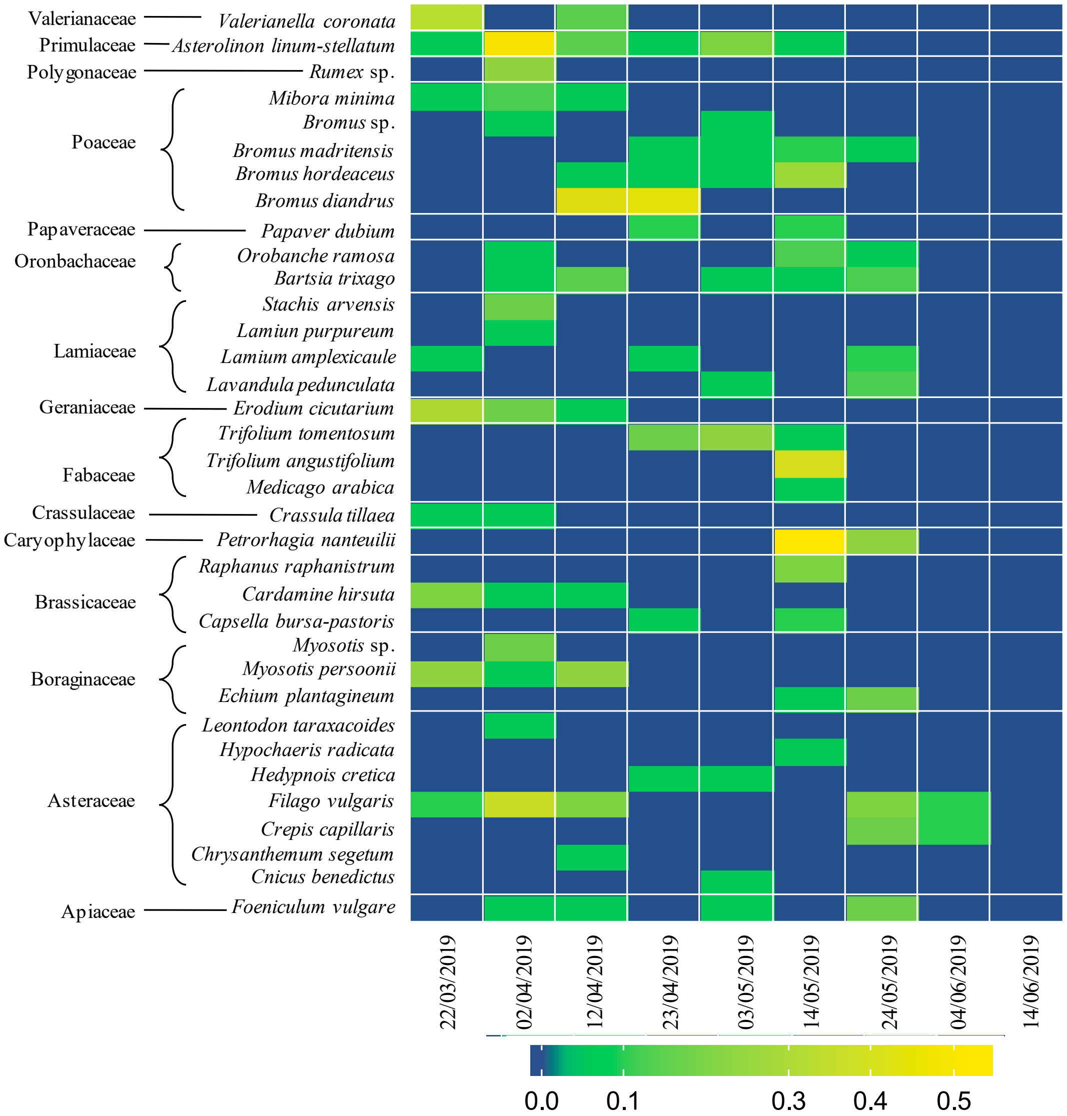

Figure S10. Heatmap plot showing the percentage of plants taxa identified with <1% of coverage through the collection period (year 2019) (dark purple to yellow) in an integrated olive orchard (non-host plants). Families are indicated at the left of the plant species name.
